# Metformin use and cardiovascular outcomes after acute myocardial infarction in patients with type 2 diabetes: a cohort study

**DOI:** 10.1186/s12933-019-0972-4

**Published:** 2019-12-09

**Authors:** Daniel I. Bromage, Tom R. Godec, Mar Pujades-Rodriguez, Arturo Gonzalez-Izquierdo, S. Denaxas, Harry Hemingway, Derek M. Yellon

**Affiliations:** 1 The Hatter Cardiovascular Institute, University College London, 67 Chenies Mews, London, WC1E 6HX UK; 2 School of Cardiovascular Medicine and Sciences, King’s College London British Heart Foundation Centre of Excellence, James Black Centre, 125 Coldharbour Lane, London, SE5 9NU UK; 3 The London School of Hygiene & Tropical Medicine, Keppel St, London, WC1E 7HT UK; 4 Leeds Institute of Health Sciences, University of Leeds, Clarendon Way, Leeds, LS2 9JL UK; 5 Institute of Health Informatics, University College London, 222 Euston Road, London, NW1 2DA UK; 6 Health Data Research UK London, University College London, 222 Euston Road, London, NW1 2DA UK; 7 The National Institute for Health Research University College London Hospitals Biomedical Research Centre, University College London, 222 Euston Road, London, NW1 2DA UK

**Keywords:** Acute myocardial infarction, Cardioprotection, Cohort studies, Metformin, Outcomes, Type 2 diabetes

## Abstract

**Background:**

The use of metformin after acute myocardial infarction (AMI) has been associated with reduced mortality in people with type 2 diabetes mellitus (T2DM). However, it is not known if it is acutely cardioprotective in patients taking metformin at the time of AMI. We compared patient outcomes according to metformin status at the time of admission for fatal and non-fatal AMI in a large cohort of patients in England.

**Methods:**

This study used linked data from primary care, hospital admissions and death registry from 4.7 million inhabitants in England, as part of the CALIBER resource. The primary endpoint was a composite of acute myocardial infarction requiring hospitalisation, stroke and cardiovascular death. The secondary endpoints were heart failure (HF) hospitalisation and all-cause mortality.

**Results:**

4,030 patients with T2DM and incident AMI recorded between January 1998 and October 2010 were included. At AMI admission, 63.9% of patients were receiving metformin and 36.1% another oral hypoglycaemic drug. Median follow-up was 343 (IQR: 1–1436) days. Adjusted analyses showed an increased hazard of the composite endpoint in metformin users compared to non-users (HR 1.09 [1.01–1.19]), but not of the secondary endpoints. The higher risk of the composite endpoint in metformin users was only observed in people taking metformin at AMI admission, whereas metformin use post-AMI was associated with a reduction in risk of all-cause mortality (0.76 [0.62–0.93], P = 0.009).

**Conclusions:**

Our study suggests that metformin use at the time of first AMI is associated with increased risk of cardiovascular disease and death in patients with T2DM, while its use post-AMI might be beneficial. Further investigation in well-designed randomised controlled trials is indicated, especially in view of emerging evidence of cardioprotection from sodium-glucose co-transporter-2 (SGLT2) inhibitors.

## Background

In acute myocardial infarction (AMI), early reperfusion by primary percutaneous coronary intervention (PPCI) is the most effective strategy for reducing infarct size and improving clinical outcome [1, 2]. However, 30 day all-cause mortality following PPCI remains significant and strategies to further improve outcomes are essential [3]. This mortality might be partly explained by the injury inflicted by the therapeutic restoration of blood flow, known as ischaemia–reperfusion injury (IRI), which may account for up to 50% of final infarct size [4–7].

Diabetes mellitus is a major risk factor for coronary heart disease (CHD), and patients with diabetes and CHD have poor clinical outcomes [8, 9]. Metformin, an oral antidiabetic of the biguanide class, exerts its effect by increasing gluconeogenesis and increasing peripheral glucose uptake. The UK Prospective Diabetes Study (UKPDS) was a large randomised, multicentre trial of glycaemic therapies in patients with newly diagnosed type 2 diabetes mellitus (T2DM) [8]. It showed lower risk of AMI in metformin users than in participants on diet therapy alone. Furthermore, all-cause and cardiovascular mortality was lower in metformin users than in sulfonylurea and insulin users, despite achieving similar glycaemic control. However, the role of metformin in reducing cardiovascular disease remains controversial, with no cardiovascular outcomes achieving statistical significance in a recent meta-analysis [10].

Nonetheless, the potential for metformin to confer acute cardioprotection in AMI is well established in the pre-clinical literature [11–15]. It has been hypothesized that the favorable effects of pre-treatment with metformin in patients with AMI relate to cardioprotection against IRI, independent of its hypoglycaemic actions [14]. However, to our knowledge, no studies have investigated outcomes after AMI in diabetic patients pre-treated with metformin, with the exception of mixed results from studies investigating the association between metformin treatment prior to ST-segment elevation AMI (STEMI) and infarct size (defined using serum biomarkers as surrogate measures) and left ventricular (LV) function [16, 17]. A mortality benefit of pre-treatment with metformin has not been demonstrated in patients with AMI and, using linked electronic health records from the CALIBER resource, which prospectively records medication status, we investigated whether metformin administration is associated with cardioprotection to these patients. Metformin is cheap and widely available, so reducing the ambiguity over its role as a cardioprotective adjunct in AMI could have global relevance [18].

## Methods

### Study design and data sources

This study used linked longitudinal electronic health records from the Clinical Practice Research Datalink (CPRD), Hospital Episode Statistics (HES) and cause-specific mortality from the Office for National Statistics (ONS) in England, which are part of the CALIBER resource (https://www.caliberresearch.org). Further details are provided in Additional file 1: Additional methods. This was a prospective cohort study and Additional file 1: Table S1 summarizes the STROBE and RECORD checklists for reporting on observational research [19, 20].

### Study population and exposure definition

The study included all available patients with T2DM experiencing their first fatal or non-fatal AMI (either STEMI or non-STEMI) between January 1998 and October 2010. Diagnoses of T2DM and AMI were identified in CPRD and HES using EHR-derived phenotypes [21, 22]. Only the earliest recorded AMI across all data sources was considered [21, 23, 24]. Eligibility criteria for study inclusion were no history of AMI prior to the study start date, a minimum of 1 year of follow-up since practice registration and since the date on which the data from their CPRD practice was deemed to be of acceptable quality, and be 18 years of age or above at the time of AMI admission. Metformin exposure status at AMI admission was defined according to whether the patient had at least one prescription within 6 months before the incident AMI. After AMI, if repeat prescriptions were at least 6 monthly, patients were defined as having ongoing drug exposure during follow-up. This was used as a time-updated covariate in survival analysis.

### Baseline characteristics

To account for recent diabetes severity, it was determined whether a patient was prescribed insulin in 6 months prior to hospital admission. Glycosylated haemoglobin was included, when it was recorded within a year prior to admission, as a marker of glycaemic control. For each patient, data on diagnosed co-morbidities and cardiovascular risk factors were identified in CPRD, HES and ONS [25]. Further details are provided in Additional file 1: Additional methods.

### Study endpoints and follow-up

The primary endpoint was a major adverse cardiac event (MACE), defined as a composite of AMI requiring hospitalisation, stroke and cardiovascular mortality. The secondary endpoints were hospitalisation for heart failure (HF) and all-cause mortality. An a priori subgroup analysis included only survivors of AMI at 30 days post-index AMI (i.e. those who left the GP practice, were ‘lost to follow-up’ or had an event within 30 days post-AMI were excluded), to distinguish between acute and chronic effects of metformin after AMI. Further details are provided in Additional file 1: Additional methods.

### Statistical analyses

Demographic and baseline characteristics of the study population with and without metformin were compared using the Pearson Chi square test for categorical variables and Student t test for continuous variables. The cumulative probability of each endpoint was calculated using Kaplan–Meier methods and the log rank test was used to examine differences between the treatment groups. Multivariable Cox proportional hazard regression was used to estimate hazard ratios for the effect of metformin use at AMI admission, adjusted for covariates where P < 0.2 in the univariable analysis or their inclusion in the models resulted in a change of the estimate of the exposure of > 10%, as well as the a priori well established cardiovascular risk factors age, sex, BMI and smoking status at time of index AMI. Further details are provided in Additional file 1: Additional methods.

### Sensitivity analyses

Sensitivity analyses were performed to examine the effect of ongoing metformin use, which has previously been associated with benefit [10]. To do so, we compared patients on metformin at the time of index AMI who had no subsequent prescriptions with those who were never prescribed metformin or had their first prescription after 6 months post-AMI, at which point they were censored). Patients with a metformin prescription within 6 months after first AMI were excluded from this analysis. We also analysed time-varying metformin use for patients who did not have a metformin prescription at AMI admission and within 6 months of AMI, for comparison with previous studies. As an alternative approach, we conducted a propensity score analysis. Propensity scores (the conditional probability that a subject received metformin) were predicted for each subject using baseline covariates in a logistic regression model. All available baseline covariates were considered, but only those that showed evidence of an association with the outcome were used in the final model [26, 27]. The propensity scores were assessed in each treatment group to ensure balance. Details of the composition of the propensity score model is given in Additional file 1. The estimated propensity scores were then used to create inverse probability weights for each subject [28]. These weights were used in a Cox proportional hazards regression model to estimate the treatment group effect. Further sensitivity analyses are described in Additional file 1: Additional methods.

## Results

### Metformin use and patient baseline characteristics

**Fig. 1:**
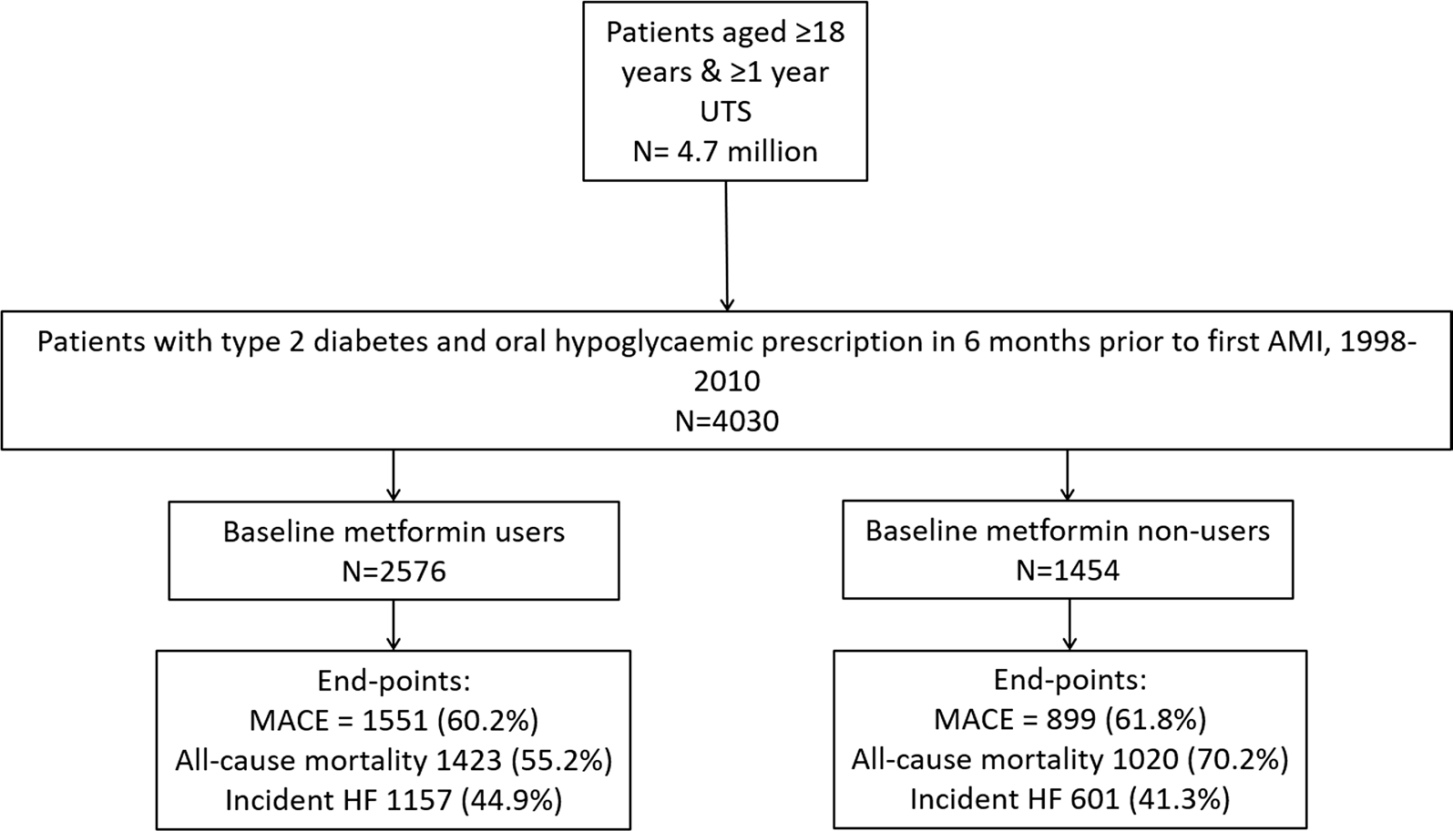
Study flow diagram. MACE, major adverse cardiac events, defined as a composite of AMI requiring hospitalisation, stroke and cardiovascular mortality. UTS, up to standard; AMI, acute myocardial infarction; HF, heart failure

Out of 4,703,682 patients in CALIBER, we identified 4030 eligible patients with T2DM and no history of HF or AMI, who had an incident AMI during the study period (Fig. 1). Of them, 2576 (63.9%) were prescribed metformin at the time of their AMI while 1454 (36.1%) received an alternative hypoglycaemic drug. The median follow-up time from first AMI (index event) to either death, leaving their GP practice or last date of data collection was 343 (IQR: 1–1436) days.

Patient baseline characteristics according to exposure group are shown in Table 1. The mean age at index AMI admission was lower for metformin users than for those taking an alternative hypoglycaemic drug (71.3 and 76.1 years, respectively), and there was a lower proportion of women (38.9% vs. 42.9%). Overall, co-morbidities and cardiovascular risk factors were similar between the two treatment groups (Table 1). Although more patients in the metformin group had a raised BMI and history of smoking, fewer had a history of peripheral arterial disease (PAD) and abdominal aortic aneurysm (AAA). Importantly, there was no clinically meaningful difference in glycaemic control, indicated by the level of HbA1c, between the two groups (metformin 61.5 mmol/mol vs. other hypoglycemics 59.0 mmol/mol, median time of measurement 96 (IQR 46–168) days prior to index AMI). More patients on metformin had recently been prescribed insulin (17.1% vs. 7.8%). Use of other oral hypoglycaemic medication was similar except for sulphonylureas, which were unsurprisingly higher in the non-metformin group (50.9% vs. 96.3%).

**Table 1:** Baseline patient characteristics by metformin status, in patients with at least one prescription in 6 months prior to admission with first acute AMI

	Metformin (N = 2576, 63.9%)	Other drug (N = 1454, 36.1%)
Age (years), mean (SD)**	71.29 (11.2)	76.10 (10.9)
Female, n (%)*	1001 (38.9)	624 (42.9)
IMD, n (%)		
< 8.5 (Least deprived)	431 (16.8)	252 (17.4)
8.5– < 34.18	1657 (64.6)	938 (64.8)
≥ 34.18 (Most deprived)	478 (18.6)	258 (17.8)
BMI (kg/m^2^), mean (SD)**		
< 18.5	23 (0.9)	27 (2.0)
18.5– < 25	490 (19.8)	388 (29.0)
25– < 30	912 (36.8)	535 (40.0)
30– < 35	703 (28.4)	275 (20.5)
≥35	352 (14.2)	115 (8.6)
Smoking status, n (%)*		
Never-smoker	1104 (44.6)	688 (50.4)
Ex-smoker	1011 (40.8)	493 (36.1)
Current smoker	363 (14.7)	183 (13.4)
Hypertensive, n (%)*	2186 (84.9)	1178 (81.0)
Systolic blood pressure (mmHg), mean (SD)*	140.50 (23.6)	138.38 (21.8)
HbA1c (mmol/mol), mean (SD)**	61.48 (17.39)	58.99 (17.16)
Diabetes medication, n (%)		
Sulphonylureas, n (%)**	1310 (50.9)	1400 (96.3)
Thiazolidinediones, n (%)	217 (8.4)	146 (10.0)
Acarbose, n (%)	69 (2.7)	37 (2.5)
DPP4 inhibitors, n (%)*	8 (0.3)	13 (0.9)
GLP1 agonists, n (%)	3 (0.1)	2 (0.1)
Meglitinides, n (%)	21 (0.8)	15 (1.0)
Insulin, n (%)**	440 (17.1)	113 (7.8)
HDL serum cholesterol (mmol/L), mean (SD)	1.18 (0.4)	1.20 (0.4)
Total serum cholesterol (mmol/L), mean (SD)**	4.43 (1.2)	4.58 (1.3)
History of cardiovascular disease		
HF, n (%)*	652 (25.3)	432 (29.7)
CHD, n (%)	1064 (41.3)	593 (40.9)
Ischaemic stroke, n (%)	89 (3.5)	49 (3.4)
Stroke, n (%)	338 (13.1)	198 (13.6)
TIA, n (%)	218 (8.5)	135 (9.3)
PAD, n (%)*	472 (18.3)	307 (21.1)
AAA, n (%)*	441 (17.1)	292 (20.1)

Patients with missing data were: 16 for IMD score, 210 for BMI, 188 for smoking status, 91 for systolic blood pressure, 1032 for HbA1c, 988 for HDL cholesterol, and 382 for total cholesterol

*AMI* acute myocardial infarction, *BMI* body mass index, *IMD* index of multiple deprivation, *HbA1c* haemoglobin A1c, *DPP* dipeptidyl peptidase, *GLP* glucagon-like peptide, *HDL* high density lipoprotein, *HF* heart failure, *CHD* coronary heart disease, *NOS* not otherwise specified, *TIA* transient ischaemic attack, *PAD* peripheral arterial disease, *AAA* abdominal aortic aneurysm

* P < 0.05 ** P < 0.001, from tests of difference using t tests for continuous variables and Chi squared tests for categorical variables

### Associations between metformin use at AMI admission and endpoints

In total, 2450 patients had a MACE endpoint during the study period, 1551 (60.2%) in metformin users at the time of their AMI and 899 (61.8%) in non-users (Table 2). The unadjusted analysis showed no statistically significantly difference between metformin user groups at hospital admission for AMI (HR 0.95 [0.88–1.03], P = 0.239, Table 2). In adjusted models, there was strong evidence of an association with the composite endpoint amongst metformin users, but the HR was small (HR 1.09 [1.01–1.19], P = 0.034, Table 2).

**Table 2:** Associations between metformin use at hospital admission for AMI and MACE over median follow-up of 343 (IQR: 1–1436) days

	Number of patients experiencing event (%)		Unadjusted		Adjusted^a^	
	Metformin (N = 2576)	Other (N = 1454)	Hazard ratio (95% CI)	P value	Hazard ratio (95% CI)	P value
Primary endpoint						
MACE (composite of cardiovascular mortality, AMI and stroke)	1551 (60.2)	899 (61.8)	0.95 (0.88–1.03)	0.239	1.09 (1.01–1.19)	0.034
Components of primary endpoint						
Cardiovascular mortality	894 (34.7%)	584 (40.2%)	0.84 (0.76–0.93)	0.001	1.06 (0.96–1.17)	0.275
AMI	807 (31.3%)	433 (29.8%)	1.01 (0.90–1.14)	0.887	1.06 (0.94–1.20)	0.363
Stroke	294 (11.4%)	195 (13.4%)	0.84 (0.70–1.00)	0.051	0.99 (0.82–1.19)	0.904
Secondary endpoints						
All-cause mortality	1423 (55.2%)	1020 (70.2%)	0.77 (0.71–0.83)	< 0.001	0.97 (0.89–1.04)	0.395
HF hospitalisation	589 (22.9)	313 (21.5)	1.01 (0.88–1.16)	0.918	1.13 (0.98–1.30)	0.092

*AMI* acute myocardial infarction, *HF* heart failure

^a^Adjusted for: age at index AMI, sex, smoking status, BMI, prior insulin use, total serum cholesterol, previous stroke, previous TIA

The median time to outcome diagnosis was 55 (IQR 0–396) days. Sequential adjustment revealed that adjusting only for age (HR 1.10 [1.01–1.19], P = 0.033) had the largest impact on the change in the size of the hazard ratio compared to the crude estimate. Further adjustment for other baseline covariates (sex, BMI, prior insulin use, total serum cholesterol, previous stroke and previous TIA) had only small added effects (Additional file 1: Table S2). Furthermore, there was no evidence for an interaction between metformin use and AMI type (P = 0.274, Additional file 1: Table S2). The propensity score analysis showed consistent results (HR 1.13 [1.03–1.23], P = 0.006, Table 3) for the composite primary endpoint. This association was driven by an increased hazard of cardiovascular mortality (HR 1.12 [1.00–1.25], P = 0.044), which was not evident in the main analysis.

**Table 3:** Associations between metformin use at hospital admission for AMI and MACE over median follow-up of 343 (IQR 1–1436) days using propensity score analysis

Endpoint	Hazard ratio (95% CI)	P value
Primary endpoint		
MACE (composite of cardiovascular mortality, AMI and stroke)	1.13 (1.03–1.23)	0.006
Components of primary endpoint		
Cardiovascular mortality	1.12 (1.00–1.25)	0.044
AMI	1.07 (0.95–1.21)	0.273
Stroke	1.00 (0.83–1.21)	0.977
Secondary endpoints		
All-cause mortality	1.02 (0.94–1.11)	0.660
HF hospitalisation	1.18 (1.06–1.31)	0.002

Adjusted for: age at index AMI, sex, ethnicity, BMI, fasted glucose, HbA1c, smoking status, total serum cholesterol, previous HF, previous stroke, previous TIA, previous AAA, previous angina and if ever prescribed insulin prior to index AMI

*AMI* acute myocardial infarction, *HF* heart failure

With respect to the secondary endpoints, the use of metformin at the time of index AMI was associated with a trend towards increased risk of HF hospitalisation (HR 1.13 [0.98–1.30], P = 0.098, Table 2) but not all-cause mortality (HR 0.97 [0.89–1.04], P = 0.395, Table 2). However, in the propensity score analysis, this association with increased HF hospitalisation reached statistical significance (Table 3).

Subgroup analysis restricted to survivors after 30 days post index AMI showed no association between metformin use and MACE (HR 1.06 [0.95–1.17], P = 0.305, Additional file 1: Table S3).

### Sensitivity analyses

Metformin use at the time of index AMI but not afterwards, compared to no metformin use, was associated with an increased hazard of the primary composite outcome (HR 1.41 [1.28–1.56], P < 0.001, Additional file 1: Table S4). This was driven by an increase in the hazard of cardiovascular mortality (HR 1.50 [1.33–1.68], P < 0.001). Patients on metformin at the time of index AMI who had no subsequent prescriptions of this drug had an increased hazard of all-cause mortality (HR 1.31 [1.19–1.43], P < 0.001) but the association with HF hospitalisation was not statistically significant (HR 1.12 [0.98–1.45], P = 0.078, Additional file 1: Table S4).

In the analysis of time-varying metformin use post-AMI in patients who were not on metformin at the time of AMI, the hazard of MACE was similar during periods of metformin use compared to periods of non-use (HR 0.96 [0.78–1.19], P = 0.732). However, consistent with previous reports, all-cause mortality was lower during periods of metformin use (HR 0.75 [0.62–0.93], P = 0.009) in this cohort, as was the hazard of hospitalised HF, although this did not reach statistical significance (HR 0.67 [0.47–1.01], P = 0.056). There was no evidence of association with cardiovascular mortality (HR 0.88 [0.67–1.15], P = 0.345). Additional sensitivity analyses are described in Additional file 1: Additional results.

## Discussion

Based on previous basic and clinical studies, we hypothesised that metformin administration would confer cardioprotection in the setting of AMI. We interrogated linked, prospectively recorded, electronic health records of patients with T2DM experiencing their first AMI, using primary care linked to hospital admission and mortality data. This is the first study investigating the association of outcomes after AMI in diabetic patients with metformin pre-treatment. We observed an older cohort, higher rates of previous cardiovascular disease and worse outcomes compared to other studies of AMI in diabetic patients [29, 30], indicating that ours is a high-risk cohort.

### Metformin use at time of AMI

Among over 4000 patients identified, the majority were receiving metformin at the time of index AMI, which was associated with worse outcomes with respect to the composite endpoint compared to patients on alternative oral hypoglycaemic agents. This which was driven by cardiovascular mortality and was the case despite similar glycaemic control in the two treatment groups, albeit it with higher rates of insulin prescription in the metformin group. This association was not evident in patients who survived the first month post index AMI, suggesting an acute, non-lasting effect. Interestingly, these findings only applied to patients taking metformin at the time of AMI, whereas post-AMI metformin use was associated with similar or reduced risk (and attenuated the strength of association in the primary analysis), in keeping with previous reports [31]. Taken together and acknowledging potential bias associated with observational studies (including a more severe diabetes phenotype as indicated by a higher use of insulin in metformin users), these findings challenge reports of an acute infarct-sparing role for metformin.

With respect to cardioprotection at the time of AMI, several preclinical studies have demonstrated acute infarct size reduction and improved cardiac function with metformin [11–15]. However, a recently published pre-clinical study of post-conditioning with metformin in pigs found no difference in infarct size or LV function compared to vehicle [32]. Human studies have used biomarkers as a surrogate of infarct size in the context of STEMI [16, 17]. A study of 660 diabetic patients with STEMI showed a reduction of infarct size (according to serum biomarkers) in the metformin group, although the comparator group were treated with diet alone, which may have exaggerated the effect [16]. A propensity score matched analysis of 493 diabetic patients with STEMI showed no association between metformin and infarct size or LV function, although no information on drug treatment of diabetes in the control group was provided [17]. In an analogous, randomized trial of oral metformin, started after primary percutaneous coronary intervention for STEMI in patients *without* diabetes, no benefit was seen on LV function, MACE, all-cause mortality, or new-onset diabetes [33, 34].

Our data also applies to patients with non-ST-segment elevation AMI (NSTEMI). Despite worse outcomes than patients presenting with STEMI [35, 36], there is no evidence regarding the use of metformin in this context. We report an association between metformin use at the time of index AMI, including NSTEMI, and worse outcomes. This was unexpected and justifies further investigation.

We were unable to investigate the reasons for the association with worse outcomes in patients taking metformin at the time of index AMI. Traditionally, metformin is stopped on admission in these patients, due to concerns over lactic acidosis. However, there is a paucity of evidence for this and guidelines from the European Society of Cardiology (ESC) no longer recommend its routine cessation [37–40]. Our findings may also relate to the metformin group being a sicker cohort with worse diabetic phenotypes as indicated by a higher use of insulin in metformin users. In critically ill patients there is a risk of hypoglycaemia-related morbidity associated with intensive glucose control with intravenous insulin [41, 42], although we did not have data on acute glucose control in our study. In pre-clinical studies, increased myocardial rupture after AMI has been observed with metformin, which has been attributed to increased AMPK-MTOR/PGC-1α-mediated cardiomyocyte autophagy, but we were unable to assess this [43].

### Metformin use post-AMI

Despite these findings, metformin treatment has been shown to be advantageous in several pre-clinical and clinical studies of various cardiovascular diseases [44–47]. Beneficial effects on mortality have been reported in patients started on metformin *after* AMI, independent of its hypoglycaemic actions [31]. This is typically attributed to the prevention of adverse ventricular remodelling [48, 49]. However, these potential effects remain controversial and a recent meta-analysis of randomised trials indicated a non-statistically significant reduction of risk for most outcomes in metformin users, compared to non-users [10]. In the present study, metformin use post-AMI was associated with a lower hazard ratio of MACE, which may support its use in patients at increased risk of cardiovascular disease.

### Clinical relevance and further work

The ESC advocate the use of metformin, despite limited evidence, because of potential safety and economic benefits [50]. The present study is consistent with its use in patients with T2DM but the association with worse outcomes among patients on metformin at the time of their AMI suggests that further investigation in well-designed randomised controlled trials is indicated, especially in view of the evidence and availability of alternatives. Although de novo randomised controlled trials of metformin are unlikely, the present study supports recent assertions that alternative approaches may comprise the inclusion of metformin in factorial trials and full publication of cardiovascular outcome data from previous trials of metformin [10].

### Strengths and limitations

The strengths of this study include, first, the use of three linked electronic data sources to maximise the ascertainment of outcomes. In addition, medication status and baseline characteristics were recorded prospectively, *prior* to AMI, limiting the possibility of recall bias. Second, the longitudinal study design and the analysis of metformin as a time-varying covariate that consider periods of use and non-use during follow-up. Third, we included patients on alternative hypoglycemics as the control group. This contrasts with similar studies that use a non-diabetic or untreated diabetic patient control arm and risk confounding due to difference in baseline characteristics. The results from the propensity score analysis were consistent, which increases confidence in our findings.

This study has the limitations of observational data, including potential bias because of unmeasured confounding factors (e.g. reperfusion status and indication bias). Therefore, we can only report associations and not causal relationships, and any results must be interpreted with caution, especially in view of clinical experience and economic indications for metformin use. Furthermore, although we accounted for HbA1c and prior insulin use, we were unable to adjust for other indicators of quality of diabetes management, including microvascular and other macrovascular complications, as these data were unavailable. However, the baseline characteristics of patients in our cohort were similar to each other and to those reported elsewhere [16]. Further strengths and limitations are addressed in Additional file 1: Additional discussion.

## Conclusions

Acknowledging the limitations of observational studies, this study suggests that metformin use at the time of AMI is associated with increased risk of cardiovascular disease and death in patients with T2DM, while its use post-AMI might be beneficial. This may relate to the metformin group being a sicker cohort with worse diabetic phenotypes. However, evidence for metformin in diabetic patients at high cardiovascular risk is mixed and resolving this is central to extracting maximum benefit from this cheap and widely available drug.

## Supplementary information

**Additional file 1.** Supplementary material.

## Data Availability

As specified in data sharing agreements, raw datasets are not available. The data that support the findings of this study are available from CPRD (https://www.cprd.com/) but restrictions apply to the availability of these data, which were used under license for the current study, and so are not publicly available.

## Abbreviations

AAA: abdominal aortic aneurysm
AMI: acute myocardial infarction
BMI: body mass index
CHD: coronary heart disease
CPRD: Clinical Practice Research Datalink
DPP: dipeptidyl peptidase
ESC: European Society of Cardiology
GLP: glucagon-like peptide
HbA1c: haemoglobin A1c
HDL: high density lipoprotein
HES: Hospital episode statistics
HF: heart failure
IMD: index of multiple deprivation
IRI: ischaemia reperfusion injury
LV: left ventricular
ONS: Office for National Statistics
PAD: peripheral arterial disease
PPCI: primary percutaneous coronary intervention
SGLT2: sodium glucose co-transporter-2
STEMI ST: ST-segment elevation myocardial infarction
T2DM: type 2 diabetes mellitus
TIA: transient ischaemic attack
