# Supplementary material for: Metformin use and cardiovascular outcomes after acute myocardial infarction in patients with type 2 diabetes: a cohort study
Source: Cardiovasc Diabetol. 2019 Dec 9;18:168. doi: 10.1186/s12933-019-0972-4 (PMC6900858; doi:10.1186/s12933-019-0972-4)
Supplement: Supplementary file 1 — Additional file 1. Supplementary material. [file 12933_2019_972_MOESM1_ESM.docx]

Additional methods

*Study design and data sources*

The CPRD is a primary care database containing longitudinal anonymized patient records from general practices for approximately 7% of the UK population (11.3 million patients).(1) Patients are broadly representative of the UK population in terms of age, sex and ethnicity. Around 45% of primary care practices contributing to the CPRD consented to data linkage of individual patient records with other data sources.(2) ONS provides cause-specific mortality and HES provides diagnostic codes, using the tenth revision of the International Classification of Diseases (ICD), related to all elective and emergency hospital admissions across all National Health Service hospitals in England. Linkage of the CPRD to HES and ONS allows longitudinal follow-up of patients after their incident AMI, and was done by a Trusted Third Party (NHS Digital), based on the patient NHS number (a ten-digit unique identifier assigned at first interaction with the healthcare system), date of birth, patient postcode and sex.

Study population and exposure definition

In each data source AMI and T2DM were defined using phenotyping algorithms extensively used and validated for cardiovascular research (3, 4) in CALIBER (available online, https:// www.caliberresearch.org/portal/).(5-7) EHR-derived phenotypes consist of Read, ICD-9 and -10 and Office of Population Censuses and Surveys (OPCS) codes.(6, 8)

Baseline characteristics

Data on diagnosed co-morbidities and cardiovascular risk factors included sex, age, smoking status, Index of Multiple Deprivation (IMD, an area-based socioeconomic deprivation indicator that is defined elsewhere (9)), total and HDL serum cholesterol, body mass index (BMI), past medical history of cardiovascular diseases, and diagnosed hypertension. Risk factors had previously been defined and phenotyping algorithms that include Read and ICD-10 codes are available online (https:// www.caliberresearch.org/portal/phenotypes).(6, 7, 9, 10) To define baseline variables, including smoking status, cholesterol, blood pressure and weight, we used the closest recorded measure to the date of entry (i.e. date on which all eligibility criteria were met), amongst measurements recorded within 1 year prior to study entry.

Study endpoints and follow-up

Endpoints were ascertained using Read codes (version 2) in CPRD, ICD-10 codes in HES and ICD-10 or ICD-9 codes in ONS and the phenotyping approach has been previously described and validated (www.caliberresearch.org/portal/).(5-7, 9-11) To define incident AMI and avoid misclassification by recurrent consultation for the same episode, only AMI diagnoses recorded after at least one month from the date of the index AMI were considered.(5) Other components of the primary outcome were considered from the day of the index AMI. The start of follow-up was the earliest date on which all inclusion criteria were met. It ended on the earliest of study endpoint occurrence, date of GP practice deregistration (i.e. ‘lost to follow-up’), death from any cause or last date of data collection in the practice.

Statistical analyses

Normality of distribution was assessed using the Shapiro-Wilks test.

Multiple imputation using chained equations was used to account for missing covariate data and 40 imputed datasets were generated.(12, 13) Risk factor data appeared to be missing at random after adjusting for major confounders (e.g. age, sex, smoking status, BMI and blood pressure). Hence, multiple imputation was implemented using the *‘mi’ a*lgorithm in the STATA software version 14.2, to replace missing values in risk factor variables.(12, 13) Multiple imputation using chained equations was used to impute missing data with 40 imputed datasets generated. Imputation models included all the baseline covariates used in the main analysis (sex, age, hypertension, smoking status, total cholesterol, systolic blood pressure, previous CHD, previous HF, previous stroke, previous TIA, previous PAD, previous AAA, previous prescribed insulin, and HbA1c). Non-normally distributed variables (HbA1c) were log-transformed for imputation. The Nelson-Aalen estimate of hazard for each separate endpoint was also included. Plausibility of imputations for all covariates was checked by comparing plots of the distribution of recorded and imputed values(14). Estimates were obtained from analyses on each of the 40 datasets, then combined using Rubin’s rules.

The proportional hazards assumption for each variable in the final adjusted models were evaluated by visually examining the Schoenfeld residuals and using a Schoenfeld residual-based test. STATA version 14.2 (StataCorp LP, College Station, USA) was used for all analyses.

Sensitivity analyses

Variables contributing to the propensity score were age at index AMI, sex, ethnicity, BMI, fasted glucose, HbA1c, smoking status, total serum cholesterol, previous HF, previous TIA, previous AAA, previous angina and if ever prescribed insulin prior to index AMI. Further sensitivity analyses included, first, a complete-case analysis including only patients with complete covariate data to assess the effect of missingness. Second, we repeated the primary analysis relaxing the assumption of independence of individuals within a GP practice, by calculating cluster-robust standard errors to take account of any differences between practices with respect to patient management and diagnosis. Finally, incident HF (first recorded event in primary care or during hospitalisation) was analysed after excluding patients with pre-existing HF.

Additional results

In the analysis including only patients with complete covariate data to assess the effect of missing data (n=3,444, **Table S5**), the increased hazard of MACE among patients taking metformin at the time of index AMI persisted (HR 1.32 [1.17-1.49], P<0.001). Similarly, there was no difference in findings when cluster-robust standard errors were used to take account of heterogeneity between practices (HR 1.09 [1.01-1.19], P=0.032, **Table S5**). Finally, in secondary analyses, the increase in the hazard of HF amongst metformin users at index AMI persisted when patients with a history of HF were excluded (HR 1.28 [1.13-1.45], P<0.001, **Table S6**).

Additional discussion

Strengths & limitations

The baseline characteristics of the study population reflect patient populations seen in UK clinical practice. We included both STEMI and NSTEMI to provide a broader description of the role of metformin in patients with AMI. Finally, the sensitivity analyses support the main finding that metformin use at the time of AMI was associated with worse outcomes in diabetic patients.

With respect to limitations, we were unable to account for reperfusion status, although it may be assumed that patients were predominantly reperfused, following clinical guidelines. We are also unable to consider the infarct size as an additional endpoint, as imaging and biomarker data were not available. Furthermore, details of drug treatment, including metformin dose and acute treatment, were not available.

In our study, we used prescribed metformin in primary care as a proxy for metformin use. Information on prescriptions issued to patients in CPRD is complete but patient adherence might decrease over time. Six-month windows following prescription were used to define drug exposure to allow some spill-over and ensure events were not incorrectly attributed to the unexposed group. While acknowledging this could bias against metformin use, the use of time-updated analysis (i.e. considering periods of use and non-use) and subgroup analyses considering patients who received medication before, during and/or after the index AMI, allowed greater accuracy in estimations and a more in-depth examination of the relationship between metformin and cardiovascular risk. Furthermore, the median time from last prescription to primary event was 25 (11-67) days, so most events occurred well inside the 6 months window and were correctly attributed. In the sensitivity analyses, we were unable to account for the reasons for metformin cessation. Nonetheless, the findings are in keeping with the overall analysis.

GP practices are self-selecting with respect to their contribution to CPRD. Nonetheless, contributing practices are considered representative of the UK population. Recent analyses comparing CPRD to the UK census showed similar distribution of patients in terms of age and sex.(15) Therefore, our population would be representative of the UK population, and results generalizable to other GP practices.

Additional tables

**Table S1.** STROBE and RECORD checklists.

|  | **Item No.** | **STROBE items** | **Location in manuscript** | **RECORD items** | **Location in manuscript** |
| --- | --- | --- | --- | --- | --- |
| **Title and abstract** | | | | | |
|  | 1 | (a) Indicate the study’s design with a commonly used term in the title or the abstract (b) Provide in the abstract an informative and balanced summary of what was done and what was found | Title and abstract (p1-2) | RECORD 1.1: The type of data used should be specified in the title or abstract. When possible, the name of the databases used should be included.  RECORD 1.2: If applicable, the geographic region and timeframe within which the study took place should be reported in the title or abstract.  RECORD 1.3: If linkage between databases was conducted for the study, this should be clearly stated in the title or abstract. | 1.1 Abstract: Methods & Results (p2)  1.2 Abstract: Methods & Results (p2)  1.3 Abstract: Methods & Results (p2) |
| **Introduction** | | | | | |
| Background rationale | 2 | Explain the scientific background and rationale for the investigation being reported | Introduction: All paragraphs |  |  |
| Objectives | 3 | State specific objectives, including any prespecified hypotheses | Introduction: Third paragraph |  |  |
| **Methods** | | | | | |
| Study Design | 4 | Present key elements of study design early in the paper | Methods: Study design and data sources |  |  |
| Setting | 5 | Describe the setting, locations, and relevant dates, including periods of recruitment, exposure, follow-up, and data collection | Methods: Study design and data sources; Study population and exposure definition; Study endpoints and follow-up |  |  |
| Participants | 6 | *(a) Cohort study* - Give the eligibility criteria, and the sources and methods of selection of participants. Describe methods of follow-up  *Case-control study* - Give the eligibility criteria, and the sources and methods of case ascertainment and control selection. Give the rationale for the choice of cases and controls  *Cross-sectional study* - Give the eligibility criteria, and the sources and methods of selection of participants  *(b) Cohort study* - For matched studies, give matching criteria and number of exposed and unexposed  *Case-control study* - For matched studies, give matching criteria and the number of controls per case | (a) Methods: Study design and data sourses; Study population and exposure definition; Study endpoints and follow-up  (b) Not applicable | RECORD 6.1: The methods of study population selection (such as codes or algorithms used to identify subjects) should be listed in detail. If this is not possible, an explanation should be provided.  RECORD 6.2: Any validation studies of the codes or algorithms used to select the population should be referenced. If validation was conducted for this study and not published elsewhere, detailed methods and results should be provided.  RECORD 6.3: If the study involved linkage of databases, consider use of a flow diagram or other graphical display to demonstrate the data linkage process, including the number of individuals with linked data at each stage. | 6.1 Methods: Study population and exposure definition, including reference to CALIBER portal  6.2 Reference to validated algortihms and codes given in Methods: Study population and exposure definition |
| Variables | 7 | Clearly define all outcomes, exposures, predictors, potential confounders, and effect modifiers. Give diagnostic criteria, if applicable. | Methods: Study population and exposure definition; Baseline characteristics | RECORD 7.1: A complete list of codes and algorithms used to classify exposures, outcomes, confounders, and effect modifiers should be provided. If these cannot be reported, an explanation should be provided. | Methods: Study population and exposure definition; Baseline characteristics, including references to previous publications and CALIBER portal |
| Data sources/ measurement | 8 | For each variable of interest, give sources of data and details of methods of assessment (measurement).  Describe comparability of assessment methods if there is more than one group | Methods: Study population and exposure definition; Baseline characteristics, including references to previous publications and CALIBER portal |  |  |
| Bias | 9 | Describe any efforts to address potential sources of bias | Methods: Statistical analyses |  |  |
| Study size | 10 | Explain how the study size was arrived at | Methods: Study population and exposure definition |  |  |
| Quantitative variables | 11 | Explain how quantitative variables were handled in the analyses. If applicable, describe which groupings were chosen, and why | Methods: Statistical analyses |  |  |
| Statistical methods | 12 | (a) Describe all statistical methods, including those used to control for confounding  (b) Describe any methods used to examine subgroups and interactions  (c) Explain how missing data were addressed  (d) *Cohort study* - If applicable, explain how loss to follow-up was addressed  *Case-control study* - If applicable, explain how matching of cases and controls was addressed  *Cross-sectional study* - If applicable, describe analytical methods taking account of sampling strategy  (e) Describe any sensitivity analyses | Methods: Statistical analyses |  |  |
| Data access and cleaning methods |  | .. |  | RECORD 12.1: Authors should describe the extent to which the investigators had access to the database population used to create the study population.  RECORD 12.2: Authors should provide information on the data cleaning methods used in the study. | 12.1 Methods: Study design and data sources; Study population and exposure definition  12.2 Methods: Study design and data sources |
| Linkage |  | .. |  | RECORD 12.3: State whether the study included person-level, institutional-level, or other data linkage across two or more databases. The methods of linkage and methods of linkage quality evaluation should be provided. | 12.3 Methods: Study design and data sources, including reference to www.caliberresearch.org and (2). |
| **Results** | | | | | |
| Participants | 13 | (a) Report the numbers of individuals at each stage of the study (*e.g.*, numbers potentially eligible, examined for eligibility, confirmed eligible, included in the study, completing follow-up, and analysed)  (b) Give reasons for non-participation at each stage.  (c) Consider use of a flow diagram | Results: Metformin use and patient baseline characteristics  Figure 1: Study flow diagram | RECORD 13.1: Describe in detail the selection of the persons included in the study (*i.e.,* study population selection) including filtering based on data quality, data availability and linkage. The selection of included persons can be described in the text and/or by means of the study flow diagram. | Methods: Study population and exposure definition  Results: Metformin use and patient baseline characteristics  Figure 1: Study flow diagram |
| Descriptive data | 14 | (a) Give characteristics of study participants (*e.g.*, demographic, clinical, social) and information on exposures and potential confounders  (b) Indicate the number of participants with missing data for each variable of interest  (c) *Cohort study* - summarise follow-up time (*e.g.*, average and total amount) | (a) Results: Metformin use and patient baseline characteristics  (b) Table 1 legend  (c) Results: Metformin use and patient baseline characteristics, paragraph 1 |  |  |
| Outcome data | 15 | *Cohort study* - Report numbers of outcome events or summary measures over time  *Case-control study* - Report numbers in each exposure category, or summary measures of exposure  *Cross-sectional study* - Report numbers of outcome events or summary measures | Results: Associations between metformin use at AMI admission and endpoints  Table 2 |  |  |
| Main results | 16 | (a) Give unadjusted estimates and, if applicable, confounder-adjusted estimates and their precision (e.g., 95% confidence interval). Make clear which confounders were adjusted for and why they were included  (b) Report category boundaries when continuous variables were categorized  (c) If relevant, consider translating estimates of relative risk into absolute risk for a meaningful time period | (a) Results: Associations between metformin use at AMI admission and endpoints  Table 2, including legend  (b) Table 1 |  |  |
| Other analyses | 17 | Report other analyses done—e.g., analyses of subgroups and interactions, and sensitivity analyses | Results: Associations between metformin use at AMI admission and endpoints; Sensitivity analyses |  |  |
| **Discussion** | | | | | |
| Key results | 18 | Summarise key results with reference to study objectives | Discussion, first paragraph |  |  |
| Limitations | 19 | Discuss limitations of the study, taking into account sources of potential bias or imprecision. Discuss both direction and magnitude of any potential bias | Discussion: Limitations | RECORD 19.1: Discuss the implications of using data that were not created or collected to answer the specific research question(s). Include discussion of misclassification bias, unmeasured confounding, missing data, and changing eligibility over time, as they pertain to the study being reported. | Discussion: Limitations |
| Interpretation | 20 | Give a cautious overall interpretation of results considering objectives, limitations, multiplicity of analyses, results from similar studies, and other relevant evidence | Discussion |  |  |
| Generalisability | 21 | Discuss the generalisability (external validity) of the study results | Discussion: Clinical implications |  |  |
| **Other Information** | | | | | |
| Funding | 22 | Give the source of funding and the role of the funders for the present study and, if applicable, for the original study on which the present article is based | Funding |  |  |
| Accessibility of protocol, raw data, and programming code |  | As specified in data sharing agreements, raw datasets are not available. Researchers can access the raw data by applying to CPRD (https://www.cprd.com/) |  | RECORD 22.1: Authors should provide information on how to access any supplemental information such as the study protocol, raw data, or programming code. |  |

**Table S2.** Hazard ratios for the associations between MACE and metformin use at hospital admission for AMI by category of key variables

| **Category** | **Sub-groups** | **Adjusted HR (95%CI)** | **P value from test for interaction with metformin use at index of AMI** |
| --- | --- | --- | --- |
| Age | <65 | 1.37 (1.10-1.72) | 0.053 |
|  | 65-<80 | 1.00 (0.89-1.13) |  |
|  | 80+ | 1.08 (0.95-1.22) |  |
| Sex | Female | 1.15 (1.02-1.31) | 0.284 |
|  | Male | 1.05 (0.95-1.17) |  |
| Smoking status | Never-smoker | 1.10 (0.98-1.23) | 0.914 |
|  | Ex-smoker | 1.07 (0.94-1.23) |  |
|  | Current-smoker | 1.13 (0.91-1.41) |  |
| BMI (kg/m^2^) | <20 | 1.01 (0.68-1.49) | 0.488 |
|  | 20-<25 | 1.05 (0.89-1.24) |  |
|  | 25-<30 | 1.19 (1.04-1.35) |  |
|  | 30+ | 1.03 (0.89-1.20) |  |
| Total serum cholesterol (mmol/L) | <3.5 | 0.99 (0.83-1.19) | 0.511 |
|  | 3.5-<5.5 | 1.10 (0.99-1.22) |  |
|  | 5.5+ | 1.16 (0.96-1.39) |  |
| Insulin prescription within 6 months | No | 1.12 (1.02-1.22) | 0.117 |
|  | Yes | 0.91 (0.72-1.16) |  |
| History stroke | No | 1.09 (1.00-1.20) | 0.966 |
|  | Yes | 1.10 (0.91-1.33) |  |
| History TIA | No | 1.08 (0.99-1.18) | 0.526 |
|  | Yes | 1.17 (0.94-1.46) |  |
| Index AMI | Non-STEMI | 1.07 (0.97-1.18) | 0.274 |
|  | STEMI | 1.21 (1.00-1.47) |  |

Adjusted for sex, age, BMI, smoking status, an insulin prescription within 6 months of index AMI, total serum cholesterol, history of stroke and history of TIA. For this analysis, multiple imputation using chained equations was used to impute missing data with 40 imputed datasets generated.

**Table S3:** Subgroup analyses of primary outcomes restricted to survivors after 30 days post index AMI

|  | **Number of patients experiencing event (%)** | | **Unadjusted** | | **Adjusted*** | |
| --- | --- | --- | --- | --- | --- | --- |
|  | **Metformin (N=1822)** | **Other (N=933)** | **hazard ratio (95% CI)** | **P value** | **hazard ratio (95% CI)** | **P value** |
| MACE (composite of cardiovascular mortality, AMI, and stroke) | 1000 (54.9) | 580 (62.2) | 0.96 (0.86-1.06) | 0.420 | 1.06 (0.95-1.17) | 0.305 |

*Adjusted for: age at index AMI, sex, smoking status, BMI, prior insulin use, total serum cholesterol, previous stroke, previous TIA. AMI, acute myocardial infarction; HF, heart failure.

**Table S4:** Primary and secondary endpoints for patients on metformin at time of AMI but no subsequent prescriptions compared to patients never on metformin

| **Outcome** | **Metformin group at time of MI**  **(n, %)**  **(N=1632)** | **Non-metformin group at time of MI (n, %)**  **(N=1415)** | **Unadjusted HR (95% CI)** | **P value** | **Adjusted HR (95% CI)** | **P value** |
| --- | --- | --- | --- | --- | --- | --- |
| **Primary outcome** | 854 (52.3) | 791 (55.9) | 1.23 (1.12-1.36) | <0.001 | 1.41 (1.28-1.56) | <0.001 |
| **CV death** | 611 (37.4%) | 526 (37.2%) | 1.24 (1.11-1.39) | <0.001 | 1.50 (1.33-1.68) | <0.001 |
| **Further MI** | 266 (16.3%) | 351 (24.8%) | 1.13 (0.96-1.32) | 0.144 | 1.16 (0.98-1.38) | 0.081 |
| **Stroke** | 122 (7.5%) | 156 (11.0%) | 0.97 (0.77-1.22) | 0.787 | 1.15 (0.90-1.47) | 0.273 |
| **Outcome** | **Metformin group at time of MI**  **(n, %)**  **(N=1632)** | **Non-metformin group at time of MI (n, %)**  **(N=1415)** | **Unadjusted HR (95% CI)** | **P value** | **Adjusted HR (95% CI)** | **P value** |
| **All-cause death** | 878 (53.8%) | 901 (63.7%) | 1.08 (0.991-1.18) | 0.081 | 1.31 (1.19-1.43) | <0.001 |
| **HF hospitalisation** | 205 (12.6%) | 261 (18.5%) | 1.07 (0.89-1.28) | 0.495 | 1.12 (0.98-1.45) | 0.078 |

*Adjusted for: age at index AMI, sex, smoking status, BMI, prior insulin use, total serum cholesterol, previous stroke, previous TIA. AMI, acute myocardial infarction; HF, heart failure.

**Table S5:** Sensitivity analyses of primary outcomes

| **Patients with complete datasets (complete case analysis)** | | | | | | |
| --- | --- | --- | --- | --- | --- | --- |
|  | **Number of patients experiencing event (%)** | | **Unadjusted** | | **Adjusted*** | |
|  | **Metformin (N=2290)** | **Other (N=1154)** | **hazard ratio (95% CI)** | **P value** | **hazard ratio (95% CI)** | **P value** |
| MACE (composite of cardiovascular mortality, AMI, and stroke) | 1381 (60.3) | 741 (64.2) | 0.97 (0.89-1.06) | 0.524 | 1.32 (1.17-1.49) | <0.001 |
| **Cluster (GP practice)-robust standard errors** | | | | | | |
|  | **Number of patients experiencing event (%)** | | **Unadjusted** | | **Adjusted*** | |
|  | **Metformin (N=2576)** | **Other (N=1454)** | **hazard ratio (95% CI)** | **P value** | **hazard ratio (95% CI)** | **P value** |
| MACE (composite of cardiovascular mortality, AMI, and stroke) | 1551 (60.2) | 899 (61.8) | 0.95 (0.88-1.03) | 0.250 | 1.09 (1.01-1.19) | 0.032 |

Adjusted for sex, age, BMI, smoking status, an insulin prescription within 6 months of index AMI, total serum cholesterol, history of stroke and history of TIA. For this analysis, multiple imputation using chained equations was used to impute missing data with 40 imputed datasets generated (except for complete case analysis).

**Table S6:** Sensitivity of HF endpoint excluding previous HF

| **Outcome** | **Metformin group at time of MI**  **(n, %)**  **(N=1924)** | **Non-metformin group at time of MI (n, %)**  **(N=1022)** | **Unadjusted HR (95% CI)** | **P value** | **Adjusted* HR (95% CI)** | **P value** |
| --- | --- | --- | --- | --- | --- | --- |
| **Incident HF** | 805 (41.8%) | 369 (36.1%) | 1.21 (1.07-1.36) | 0.002 | 1.28 (1.13-1.45) | <0.001 |

*Adjusted for: age at first MI, sex, smoking status, BMI at first MI, prior insulin use, total serum cholesterol, previous stroke and previous TIA.

References

1. Herrett E, Gallagher AM, Bhaskaran K, Forbes H, Mathur R, van Staa T, et al. Data Resource Profile: Clinical Practice Research Datalink (CPRD). Int J Epidemiol. 2015;44(3):827-36.

2. Denaxas SC, George J, Herrett E, Shah AD, Kalra D, Hingorani AD, et al. Data resource profile: cardiovascular disease research using linked bespoke studies and electronic health records (CALIBER). Int J Epidemiol. 2012;41(6):1625-38.

3. Herrett E, Bhaskaran K, Timmis A, Denaxas S, Hemingway H, Smeeth L. Association between clinical presentations before myocardial infarction and coronary mortality: a prospective population-based study using linked electronic records. European heart journal. 2014;35(35):2363-71.

4. Herrett E, George J, Denaxas S, Bhaskaran K, Timmis A, Hemingway H, et al. Type and timing of heralding in ST-elevation and non-ST-elevation myocardial infarction: an analysis of prospectively collected electronic healthcare records linked to the national registry of acute coronary syndromes. European heart journal Acute cardiovascular care. 2013;2(3):235-45.

5. Herrett E, Shah AD, Boggon R, Denaxas S, Smeeth L, van Staa T, et al. Completeness and diagnostic validity of recording acute myocardial infarction events in primary care, hospital care, disease registry, and national mortality records: cohort study. BMJ. 2013;346:f2350.

6. Dinesh Shah A, Langenberg C, Rapsomaniki E, Denaxas S, Pujades-Rodriguez M, Gale CP, et al. Type 2 diabetes and incidence of a wide range of cardiovascular diseases: a cohort study in 1.9 million people. Lancet. 2015;385 Suppl 1:S86.

7. Rapsomaniki E, Timmis A, George J, Pujades-Rodriguez M, Shah AD, Denaxas S, et al. Blood pressure and incidence of twelve cardiovascular diseases: lifetime risks, healthy life-years lost, and age-specific associations in 1.25 million people. Lancet. 2014;383(9932):1899-911.

8. George J, Rapsomaniki E, Pujades-Rodriguez M, Shah AD, Denaxas S, Herrett E, et al. How Does Cardiovascular Disease First Present in Women and Men? Incidence of 12 Cardiovascular Diseases in a Contemporary Cohort of 1,937,360 People. Circulation. 2015;132(14):1320-8.

9. Pujades-Rodriguez M, Timmis A, Stogiannis D, Rapsomaniki E, Denaxas S, Shah A, et al. Socioeconomic deprivation and the incidence of 12 cardiovascular diseases in 1.9 million women and men: implications for risk prediction and prevention. PLoS One. 2014;9(8):e104671.

10. Pujades-Rodriguez M, George J, Shah AD, Rapsomaniki E, Denaxas S, West R, et al. Heterogeneous associations between smoking and a wide range of initial presentations of cardiovascular disease in 1937360 people in England: lifetime risks and implications for risk prediction. Int J Epidemiol. 2015;44(1):129-41.

11. Koudstaal S, Pujades-Rodriguez M, Denaxas S, Gho JM, Shah AD, Yu N, et al. Prognostic burden of heart failure recorded in primary care, acute hospital admissions, or both: a population-based linked electronic health record cohort study in 2.1 million people. Eur J Heart Fail. 2016.

12. Carpenter JR, Kenward MG. Multiple imputation and its application. 1st ed. Chichester, West Sussex: John Wiley & Sons; 2013. p. p.

13. Royston P, White IR. Multiple imputation by chained equations (MICE): Implementations in STATA. Journal of Statistical Software. 2011;45(4):1-20.

14. van Buuren S. Multiple imputation of discrete and continuous data by fully conditional specification. Statistical methods in medical research. 2007;16(3):219-42.

15. Herrett E. Data Resource Profile: The Clinical Practice Research Datalink. 2014.
